# Correction: Lichterfeld et al. Porcine Nose Atrophy Assessed by Automatic Imaging and Detection of *Bordetella bronchiseptica* and Other Respiratory Pathogens in Lung and Nose. *Animals* 2024, *14*, 3113

**DOI:** 10.3390/ani15152160

**Published:** 2025-07-22

**Authors:** Hanna Lichterfeld, Sara Trittmacher, Kathrin Gerdes, Kathrin Schmies, Joaquín Miguel, Irene Galé, Alba Puigredon Fontanet, Isaac Ballarà, Krista Marie Tenbrink, Isabel Hennig-Pauka

**Affiliations:** 1 Field Station for Epidemiology, University of Veterinary Medicine Hannover, Foundation, 49456 Bakum, Germanysara.trittmacher@gmx.de (S.T.); kathrin.gerdes@tiho-hannover.de (K.G.); info-bakum@tiho-hannover.de (K.S.); 2 HIPRA, 17170 Amer, Spain; 3 HIPRA Deutschland GmbH, 40211 Düsseldorf, Germany

## Error in Figure

In the original publication [1], there was a mistake in Figure 8. Detection of *B. bronchiseptica* in the nose and the lower respiratory tract (*n* = 121) as published. The depiction of the *x*-axis and the depiction of the columns were wrong. The corrected Figure 8 appears below. The authors state that the scientific conclusions are unaffected. This correction was approved by the Academic Editor. The original publication has also been updated.

## Figures and Tables

**Figure 8:**
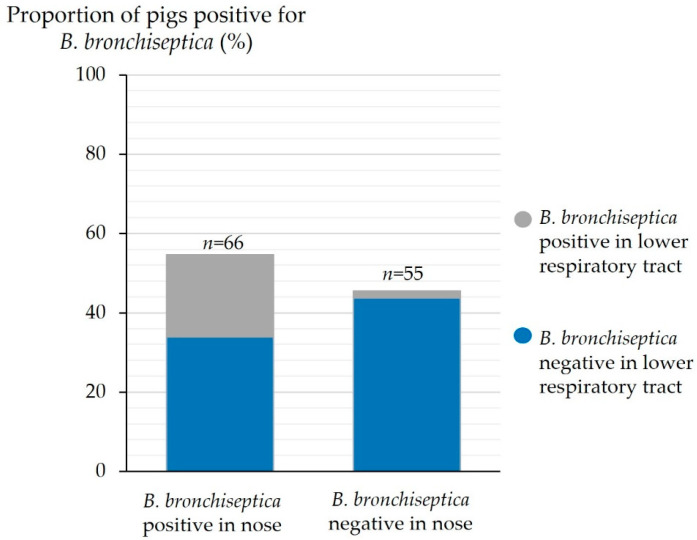
Detection of *B. bronchiseptica* in the nose and the lower respiratory tract (*n* = 121).
